# Medical Opinion and Movement

**Published:** 1909-08-21

**Authors:** 


### Medical Opinion and Movement.

At a recent meeting of la Société Médicale des Hôpitaux attention was called by Roger and Lévy-Valensi to a reaction which is of use in testing sputum in doubtful cases of Tuberculosis. This they call the albumino-reaction of sputum, and its main advantage lies in the simplicity of its application. The sputum is diluted with a little water, the contained mucus is precipitated with acetic acid, and the mixture then filtered. The filtrate is next tested for albumin by heating and adding a few drops of potassium ferrocyanide. A positive result has been obtained constantly with tuberculous sputum, but acute and chronic bronchitis gives a negative one. Albuminous exudates are found in cases of pulmonary congestion, broncho-pneumonia, and pneumonia during the acute stage, but these disappear when the inflammatory period has passed away. In the case of a slowly resolving broncho-pneumonia a negative reaction eliminates tuberculosis. The reaction is not meant to displace the usual methods for the detection of tubercle in the lungs, but is useful in being a rapid, and, in the authors' opinion, a reliable test for the absence of tubercle when its results are negative.

The therapeutic value of Lumbar Puncture in cases of cranial injury is well brought out by Savy in a case reported by him in *Le Lyon Chirurgical.* The patient was brought into hospital unconscious after a fall from a height. The appearance, a day or two later, of a subconjunctival hæmorrhage, led to the diagnosis of a fracture of the basis cranii; but no surgical interference was deemed advisable in view of the progressive amelioration of the condition of the patient, who took his discharge from hospital five weeks after the accident. It was not until three months later that the patient experienced further symptoms, consisting of weakness and rigidity of the lower limbs accompanied by constant headache. After a few weeks in bed, the patient was readmitted to hospital with characteristic meningeal symptoms—headache, sickness, constipation, delirium, a fluctuating temperature, and a well-marked Kernig's sign. In addition he complained of pain along the course of both sciatic nerves. Lumbar puncture was performed, and a collection of black, tarry blood was evacuated drop by drop. No improvement followed this first puncture, but a second and a third led to improvement. No blood or cerebro-spinal fluid was obtained at a fourth operation. The interest of the case lies in the long duration of the latent period between the accident and the onset of symptoms, and in the possibility of evacuating a still-fluid effusion from the cord so long after its production there. The author is of opinion, in view of this result, that lumbar puncture should be tried in all cases where more or less definite nervous manifestations follow a cranial injury, even when a marked interval of time has elapsed between the injury and the onset of symptoms.

The degree to which different individuals are affected by Bites from Insects is extremely variable; some persons suffer from very great pain and discomfort even from flea-bites; midge-bites make some individuals really ill; mosquito bites are painful in almost everybody. There is no specific remedy for this pain, many preparations have been tried. The following have been particularly recommended:—1. Parogenum B.P.C.; 2. Parenol liquidum B.P.C.; 3. Iodine ʒss.; petrolatum saponatum ℥j. (Moloney). Saponated petrolatum is prepared by mixing together the following: Liquid paraffin, 100 parts; oleic acid, 50 parts; alcoholic solution of ammonia containing 10 per cent. of ammonia by weight, 25 parts. A few drops of the application rubbed over a mosquito bite often removes the pain like magic, and it may also be used for other bites, such as those of bees or wasps.

We recently quoted in these columns a report upon repeated Cæsarean Section. Garipuy, in *La Presse Médicale*, has now collected a number of cases in which this operation has been performed twice. From a study of these he has formulated rules to guide the operator regarding both the proper time to intervene and the *technique* to be followed. In view of the possibility of a uterine rupture through the scar of the old incision should surgical interference be delayed until after the onset of labour, he believes that operation should take place as near term as possible. The abdominal incision should be made parallel to and a little to one side of the old scar, which then should be completely resected to guard against hernia. The first difficulty met with arises from the presence of utero-parietal, utero-omental, and utero-intestinal adhesions. Of these the last are the most troublesome, and at times resection of part of the intestines may be necessary. Utero-parietal adhesions are, however, much the most common, and if abundant will render delivery through a median incision impossible. A lateral incision will be necessary in such cases, or a hysterectomy may even have to be performed. The uterine incision, when possible, should be made parallel to and a little to one side of the former operation scar. A further difficulty may then arise from adherence of the placenta to the scar. Two circumstances render hysterectomy necessary. If there be marked thinning of the scar, a hysterectomy will prevent the dangers to which a subsequent pregnancy may give rise. The presence of a large number of adhesions, which a second operation will still further increase, render it undesirable that a further pregnancy be possible. If it be found impossible to remove the organ, the tubes should be ligatured with a view to sterilising the patient. Despite the increased difficulty of subsequent operations, the author believes, however, that hysterectomy should be performed only when the indications are especially well marked.

In cases of mixed infections the possible attenuating or intensifying effect of one microbe upon another is well known. An interesting example of an Anthrax Infection Modified and Attenuated by the Bacillus Fluorescens is reported by Dr. F. Chevrel in *Le Progrès Médical*. The case was that of a man aged 38 who developed a typical malignant pustule on the face. It was treated by cauterisation and injections of carbolic acid, and the patient was well in 10 days. Bacteriological examination showed the presence of the anthrax bacilli and also of the bacillus fluorescens in still larger quantities. A guinea-pig inoculated with discharge from the pustule only died 12 days after inoculation. This fact suggested an attenuation of the anthrax by antagonism between it and the bacillus fluorescens and led to further investigations, which fully confirmed the supposition. Mixed cultures showed rapid changes in and disappearance of the anthrax bacilli with development of the bacillus fluorescens, and by inoculation of guinea pigs with cultures of the two bacilli in different combinations, the antagonism between them was clearly demonstrated. In the course of the experiments with culture-growths, it was found that the antagonistic power of the bacillus fluorescens to the anthrax bacillus gradually diminished.

---

In *Le Progrès Médical*, Dr. J. Milhit reports the results of his observations on the Variations of the Opsonic Index in a series of cases of Enteric Fever. These cases are divided into two categories, some of them being treated simply with cold baths, while others received injections of the antityphoid serum of Chantemesse. In an ordinary case of enteric fever the opsonic index is generally found to rise steadily during the first period of the disease, and then to undergo oscillations till the temperature falls and convalescence sets in, when there is a further considerable rise in the opsonic index up to 4 or 5, followed by a gradual decline. With an injection of the serum, however, the early rise is much more sudden, attaining a maximum of 4 or 5 within 48 hours, and this is accompanied by an increased leucocytosis, an increase in the size of the spleen, an elevation of arterial tension, and a greater circulatory activity. The author points out that although this antityphoid serum does not produce the same rapid cessation of symptoms and abortion of the disease as is evidenced in diphtheria with antitoxin injections, these facts show that it stimulates in a remarkable manner the natural powers of reaction of the organism to the disease, and is to be regarded therefore as of considerable therapeutic value. The author's observations on changes in the opsonic index in the course of the disease and accompanying complications are also of interest. Relapses are associated with a fall in the index, followed by a subsequent rise, as the temperature descends again to normal. Hæmorrhages and perforations are always accompanied by rapid falls in the index. In children the opsonic index is not so well marked; it seldom attains a high figure, and does not show such wide oscillations.

At a recent meeting of the Académie des Sciences, Dr. A. Barillé reports the interesting and important observation that Pasteurisation of Milk Causes a Decomposition of the Soluble Double Salt of Calcium Carbonophosphate into the two insoluble salts of calcium carbonate and bicalcium phosphate. These salts are accordingly precipitated, and the milk is thereby impoverished in regard to its calcium content. The author points out that for infants this loss in calcium salts is a serious matter, as they play so important a rôle in the development of the skeleton and also in the gastric caseation and digestion of the milk. He suggests sterilisation of milk by means of the ultra-violet rays. A large amount of work has been recently done by French authorities on the sterilising power of the ultra-violet rays. MM. V. Henri and G. Stodel have demonstrated the bactericidal effect of these rays on milk. On the other hand, MM. Courmont and Nogier, discussing the subject at the same meeting, point out that whereas these rays rapidly sterilise a limpid fluid like water, their effect is much weaker on fluids containing colloidal substances. Water containing bacilli coli was rapidly sterilised at a distance of 30 centimetres but the addition of peptone necessitated exposure 10 times longer and at a distance 10 times nearer. Similarly it is found that the tetanus toxine is attenuated by the rays much more rapidly in a dilute solution. It seems possible, however, that with further research the ultra-violet rays may prove to be a valuable sterilising agent under certain conditions.

---

Cammidge's Reaction, the presence of which is supposed to indicate pancreatic disease, has been the subject of a good deal of research since it was first made known five years ago, but so far the substance which gives rise to the reaction has not been definitely ascertained. It will be remembered that the reaction consists essentially in the fact that the urine only gives a precipitate with phenylhydrazine after it has been boiled with an acid. Such a reaction pointed to the presence of a carbohydrate of complex nature, and Cammidge was of opinion that it was probably a pentosone. Dr. K. Smolenski has endeavoured to throw light on the question, and in a case of cancer of the stomach under his observation he found that the urine caused a deviation of polarised light to the right, reduced Fehling's solution only after heating with an acid, and gave a precipitate with phenylhydrazine only under the conditions laid down by Cammidge. These facts pointed to the presence of saccharose, and this was confirmed by further observations. The phenylosazone obtained was identical with glucosazone and the optic properties of the urine before and after heating with an acid conformed to the presence of saccharose. The addition of cane sugar to the diet is also said to have caused an increase in the intensity of the reaction of the urine. It would seem, therefore, that under certain conditions cane sugar fails to undergo the usual conversion to dextrose and levulose, and is excreted as such in the urine.

The Accuracy of the Technique of Opsonic Determination has been several times questioned by various British and foreign bacteriologists, and the method of Wright and Douglas has naturally been the most criticised, as being the most authoritative and the most largely employed of the various techniques suggested. Drs. Glynn and Cox, of Liverpool, contribute to the *Biochemical Journal* a very careful analysis of the possible sources of error, and an estimate of what inaccuracy is still unavoidable in skilled hands. The severest criticisms of opsonic technique hitherto are those of Fitzgerald, Whiteman and Strangeways, published in the "Bulletin for the Study of Special Diseases," Cambridge, in 1907; but Glynn and Cox conclude that Wright's technique is a great deal more accurate than is there suggested, and they believe they can show certain faults in method which satisfactorily account for the vitiated results obtained Adopting every precaution to ensure perfection of method and to exclude auto-suggestion, they find the average difference between each set of triple indices taken in pairs is 0.15 for tubercle and 0.17 for staphylococcus. They disagree with the critics who maintain that the technique of Wright and Douglas is useless as a means of comparing degrees of phagocytosis; but at the same time they attribute no diagnostic importance to an index between 0.8 and 1.2, even when estimated by an expert, unless the observations have been repeatedly confirmed.

---

Dr. Benjamin Moore protests against the current practice in pharmocological and therapeutic investigation of stating Dosage in Proportion to Body Weight. He points out that, in the main, an adult man of 150 lbs. weight cannot properly be given fifteen times the dose of a drug such as would suit a baby of 10 lbs., but usually about six times only. Further, small mammals such as mice and rats can take very much larger doses per unit of body weight of many tonic drugs than can rabbits, and these again than men or horses. It is to this that he ascribes the failure of atoxyl to cure hypanosomiasis in large mammals, whereas it is easily successful in rats: for the larger animals are poisoned by the drug when its proportion in the circulation is still insufficient to kill the hypanosomes. He proposes that body surface should be the property taken into account in dosage, not body weight; and for practical purposes this may be regarded as the two-third power of the body weight. In the case of substances which act by stimulation or inflammation of surfaces, such as the intestinal tract, the maximum dose is proportional to this factor. Since large animals have less intestinal and cutaneous surface per unit of weight, they can only take up proportionately less drug; and if any remedial substance is manufactured by the surface cells, they can manufacture relatively less than can smaller animals. This new rule may explain why in human therapeutics the dosage for individuals of different sizes does not rise or fall in direct proportion to the weight: the sixteen stone man may want a larger dose than one of eight stone, but does not generally require twice as much.

---

The subject of Miner's Nystagmus was brought somewhat prominently before the profession at the Sheffield meeting by the late Mr. Simeon Snell; but the condition has been known to colliery surgeons for a very long time, and not all of Mr. Snell's views are accepted by those who see many of these cases. Dr. T. H. Butler contributes to the *Ophthalmoscope* a careful account of the symptoms and causation of the disease. He points out that the nystagmus differs from ordinary nystagmus in that it gives rise to the subjective sensation of surrounding objects being in rapid movement and to vertigo. It is essentially a colliery disease, and is not found among ore miners, boiler makers, nor employees in photographic plate factories, all of whom work in semi-darkness, and some of them in positions as cramped as those of the coal miner. In the early phases of the disease nystagmus is elicited only when the eyes are turned up; it is incorrect that the movement ceases when the head is thrown back. Mr. Snell believed that acquired nystagmus is found in other trades, and collected 22 cases, several of them among compositors; but Dr. Butler is convinced that these were not in the least the same disease, and were in fact examples of asthenopia with nystagmoid movements. The disease is not more prevalent in "gassy" mines than in those free from fire damp; therefore the inhalation of gases cannot account for the symptoms. But apparently nystagmus is commoner where Davy lamps are used, that is where fire damp is especially to be feared, an apparent contradiction which the author does not explain.

As for ætiology, Dr. Butler is against the fatigue hypothesis postulated by Snell, Nieden and Dransart. When severe muscular strain does exist it is manifested, as a rule, by asthenopia, not by nystagmus. Furthermore, though the miner's position may appear cramped, in actual fact he takes good care to make himself comfortable, and does not strain his eyes at all for this reason. This theory also will not account for lateral nystagmus, for it is the elevators which are supposed to become fired by excessive use. The next hypothesis is that the nystagmus is due to excessive accommodation in a bad light; but this the author will have none of, for he says the miner does not accommodate excessively. The supposition that poor illumination accounts for the condition is more probable than the myopathic explanation; but this cannot, the author says, be the sole cause because photographic plate manufacturers do not get nystagmus. The darkness is, however, he says, a powerful adjunct in the production of nystagmus; and the stronger the lamps used, the less trouble is there among the miners from this cause. He concludes, finally, that Miner's Nystagmus—like that of disseminated sclerosis—is a cerebral symptom, the result of the collier's work which in some way not yet understood disturbs the ocular equilibrium so that rhythmic oscillations appear.

